# Genetic Variation of the Major Histocompatibility Complex (MHC Class II B Gene) in the Threatened Hume’s Pheasant, *Syrmaticus humiae*

**DOI:** 10.1371/journal.pone.0116499

**Published:** 2015-01-28

**Authors:** Weicai Chen, Yongjian Bei, Hanhua Li

**Affiliations:** 1 Natural History Museum of Guangxi, Nanning 530012, People’s Republic of China; 2 College of Life Science and Technology, Yulin Normal University, Yulin 537000, People’s Republic of China; 3 Guangxi Key Laboratory of Rare and Endangered Animal Ecology, College of Life Sciences Guangxi Normal University, Guilin 541004, People’s Republic of China; CSIRO, AUSTRALIA

## Abstract

Major histocompatibility complex (MHC) genes are the most polymorphic genes in vertebrates and encode molecules that play a crucial role in pathogen resistance. As a result of their diversity, they have received much attention in the fields of evolutionary and conservation biology. Here, we described the genetic variation of MHC class II B (MHCIIB) exon 2 in a wild population of Hume’s pheasant (*Syrmaticus humiae*), which has suffered a dramatic decline in population over the last three decades across its ranges in the face of heavy exploitation and habitat loss. Twenty-four distinct alleles were found in 73 *S. humiae* specimens. We found seven shared alleles among four geographical groups as well as six rare MHCIIB alleles. Most individuals displayed between one to five alleles, suggesting that there are at least three MHCIIB loci of the Hume’s pheasant. The *d*_N_ ⁄ *d*_S_ ratio at putative antigen-binding sites (ABS) was significantly greater than one, indicating balancing selection is acting on MHCIIB exon 2. Additionally, recombination and gene conversion contributed to generating MHCIIB diversity in the Hume’s pheasant. One to three recombination events and seventy-five significant gene conversion events were observed within the Hume’s pheasant MHCIIB loci. The phylogenetic tree and network analysis revealed that the Hume’s pheasant alleles do not cluster together, but are scattered through the tree or network indicating a trans-species evolutionary mode. These findings revealed the evolution of the Hume’s pheasant MHC after suffering extreme habitat fragmentation.

## Introduction

The Hume’s pheasant (*Syrmaticus humiae*) is considered an endangered species of the Phasianidae family, found in northeastern India, northern Myanmar, northwestern Thailand and southwestern China [1]. The population size of *S. humiae* is approximately 6,000–15,000 mature individuals [1, 2]. In India, *S. humiae* appears to be rare [3, 4]. In Myanmar, *S. humiae* may have undergone a range extension [1]. In Thailand, *S. humiae* is probably declining slowly [1]. In China, however, *S. humiae* is undergoing a decline across many of its ranges, due to heavy exploitation and habitat loss over the last three decades [5, 6]. Suitable habitat has been destroyed and severely fragmented by extensive shifting cultivation, logging and conversion to agriculture. Illegal hunting is ongoing. As a consequence, the wild population has been decreasing dramatically. Due to these severe declines in geographic range and abundance, *S. humiae* is listed as a Grade I National Protected Wildlife in China [7] as well as a globally near threatened (NT) species by the IUCN (International Union for Conservation of Nature) [2]. Although suffering a dramatic reduction, several previous studies following a neutral genetic marker (mitochondrial DNA D-Loop fragments) [8] revealed that a wild population of *S. humiae* collected from Guangxi and Guizhou Provinces did not display lower genetic diversity or significant genetic differentiation. Using RAPD (random amplified polymorphic DNA) methods to investigate a captive population of *S. humiae*, the results indicated relatively higher genetic diversity and differentiation in the captive population [9]. Neutral markers have been developed to test population structure, phylogeography and effective migration as well as determining the provenance of populations, but adaptive markers are often associated with the natural selection on genes [10, 11]. So combined neutral and selective markers can deliver more information on evolutionary history of species [12–14].

The major histocompatibility complex (MHC) genes are the most polymorphic loci in the vertebrate nuclear genome [15], and play a crucial role in the immune response against pathogens [16,17]. MHC genes have two major classes, class I and class II. The former is responsible for the recognition and binding of intra-cellular antigens, and the latter corresponds to extracellular antigens [18]. As it partially encodes the functionally important antigen-binding sites (ABS), many studies focus on the second exon of the MHC class II B locus (MHCIIB) [19]. Balancing selection is expected to act intensively on ABS, which are used to test adaptive selection in the MHC molecule. As a result, the MHC is considered a barometer for the genetic health of wild populations [20]. When carrying out conservation programs, MHC genes are therefore useful molecular markers for assessing the status of endangered populations.

In this study, we test genetic variation of part of exon 2 of the MHCIIB gene of *S. humiae*, which includes amino acid residues contained within the antigen-binding sites (ABS) of the MHC class II molecule [21]. We focus on: (i) how much diversity is observed in the MHC of *S. humiae*, and (ii) whether there is evidence of balancing selection as revealed by phylogenetic analysis, gene conversion and analysis of non-synonymous to synonymous substitution ratios.

## Materials and Methods

### Ethics statement

Sampling was approved by the Forestry Administration of Guangxi Zhuang Autonomous Region, Jinzhongshan National Nature Reserve, Guzhang Forestry Center, Longtan Nature Reserve. *S*. *humiae* is listed as Grade I National Protected Wildlife in China, and has small a population size. Therefore, most specimens came from nature reserves and local residents. Due to an edict preventing further specimen collection, specimens from local residents who had preserved tail sections prior to *S*. *humiae* being listed as a protected species were utilized. Muscle tissues were obtained from the preserved sections. Blood samples were obtained by trapping. *S*. *humiae* specimens were immediately released after the blood samples were collected. The Natural History Museum of Guangxi, Yulin Normal University, and Guangxi Normal University provided ethical approval for this study.

### Sample collection and DNA extraction

**Figure 1:**
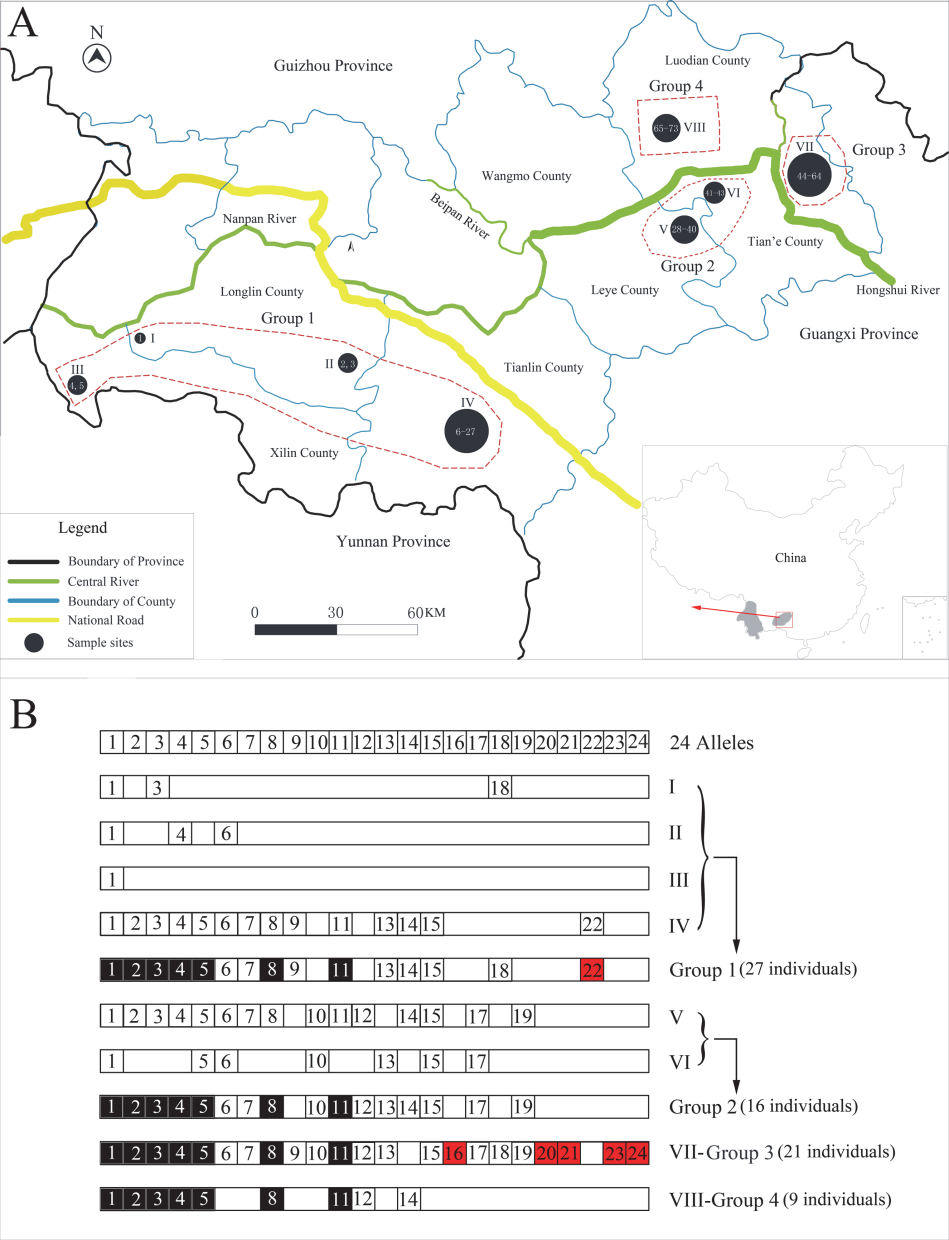
Sampling sites and allele distributions. (A) Circles indicate sample locations, among them I = 1, II = 2, III = 2, IV = 22, V = 13, VI = 3, VII = 21, and VIII = 9. Numbers indicate sample’s ID (S1 Table). Four geographical populations (group 1 to group 4) are divided according to the previous study [8]. Grey shade means the range of the Hume’s pheasant (*Syrmaticus humiae*) in China. (B) MHC class II B (MHCIIB) exon 2 allele distributions. Black boxes indicate allele sharing among groups, and red boxes indicate rare alleles.

In this study, we collected 73 *S. humiae* samples from Guangxi and Guizhou Provinces, China. Seventy-three wild individuals were obtained from six localities: Longlin County (n = 3), Xilin County (n = 2), Tianlin County (n = 22), Leye County (n = 13), Tian’e County (n = 24) and Luodian County (n = 9) (Fig. 1A; S1 Table). Among them, three blood specimens were obtained from live trapping in the field and the rest of the specimens came from muscle or skin stored in faunal collections of nature reserves or local residents. All specimens were stored in 95% ethanol and archived at Yulin Normal University, Yulin, China. Total genomic DNA was extracted using the QIAGEN DNeasy Blood & Tissue Kit according to the manufacturer’s protocol.

### PCR amplification, cloning and sequencing

In order to obtain MHCIIB exon 2 of *S*. *humiae*, we aligned seven related Phasianidae species, *Syrmaticus reevesii* (GenBank accession no.: JQ001778), *Phasianus colchicus* (AJ224345, AJ224348, HQ738665, X75406), *Gallus gallus* (HQ203710), *Chrysolophus amherstiae* (JQ001777), *Chrysolophus pictus* (JQ440366), *Crossoptilon auritum* (JQ001781, JQ001782) and *Crossoptilon crossoptilon* (JQ001779), and designed one new primer pair in conserved regions (SyF: 5’-ACCCAGCAGGTGAGGCATGTG-3’; SyR: 5’-GCTCCTCTGCACCGTGAAGG A-3’). The primer pair was situated at the borders of MHCIIB exon 2, of which the amplified region corresponded to amino acids 21 to 91 of *β* chain of human MHC class II HLA-DR [21]. PCRs were performed using 100 ng of each primer, 1µl of template DNA and 1.0 unit Hotstart-Taq DNA polymerase (Takara), 10 mM Tris-HCl, 50 mM KCl, 2.5 mM MgCl_2_, 0.2 mM of each dNTP in a final volume of 50µL. The thermal profile consisted of 5 min at 94°C, followed by 34 cycles at 94°C for 30s, 62°C for 40s, and 72°C for 30s, with a final 8 min extension at 72°C. PCR products were puriﬁed on a 1.5% agarose gel and the band corresponding to the ampliﬁed product isolated with QIAGEN QIAquick Gel Extraction Kit according to the manufacturer’s protocol. Purified PCR products were cloned into the pMD-18T vector (Takara) following manufacturer’s instructions. Fifteen to twenty clones were checked for the correct sized PCR product of 222 bp. After checking, at least 12 correctly sized products were sequenced for each individual using ABI 3730 DNA analyzer (Applied Biosytems, USA).

### Statistical analysis

All sequences were verified through homology with publicized MHCIIB alleles of other species using BLASTN (http://blast.ncbi.nlm.nih.gov/Blast.cgi) from NCBI. A new sequence variant was considered as an allele when it met the criteria summarized by Kennedy et al. [22]. The criteria are that when using DNA cloning and sequencing there have to be at least three identical clones, identified in either two separate PCRs from the same samples, or from PCRs from at least two different samples [22]. Sequence alignments were performed using the software MEGA 5 [23]. The numbers of haplotypes and nucleotide diversity (*π*) were calculated using the program DnaSP 5.0 [24]. We used the modified Nei-Gojobori/Jukes-Cantor [25] method in MEGA 5 to calculate the average rate of non-synonymous (*d*_N_) and synonymous (*d*_S_) substitution for each coding region.

Balancing selection at MHCIIB exon 2 was tested using one-tailed Z-test in MEGA 5 and compared with the likelihoods of codon-based models of sequence evolution in PAML 4.7 [26]. The Z-test was carried out for all codons, antigen-binding sites (ABS) and non antigen-binding sites (non-ABS) using 1000 bootstrapped replicates in MEGA 5. The locations of ABS and non-ABS were assumed congruent with the human class II antigen [21].

Based on the codon-based approaches, we calculated four models: M1a (neutral), assuming two site classes, with 0 < ω_0_ < 1 and ω_1_ > 1 respectively; M2a (selection), adding a third site class to M1a, with ω_2_ > 1. M7 (β) with beta distribution approximating *d*_N_ ⁄ *d*_S_ variation; M8 (β & ω) with a proportion of sites evolving with *d*_N_ ⁄ *d*_S_ > 1. The topology used in the CODEML analysis was constructed in MEGA 5, using the neighbor-joining (NJ) method with Kimura’s two-parameter evolutionary distances. Conﬁdence for the topology was evaluated using 1000 bootstrapped replicates. M1a vs. M2a and M7 vs. M8 were used to form two pairs of likelihood ratio tests (LRTs) for which the twice log likelihood difference (2△InL) was compared to the *x* ^*2*^ distribution to determine the better-fit models. If the selection models (M2a and M8) were significantly better than the neutral models (M1a and M7), we used Bayesian statistics (Bayes empirical Bayes, BEB) integrated in CODEML to identify codon sites under balancing selection [27].

Recombination events were detected using RDP 3.44 with default selections of RAP, GENECONV and MaxChi methods [28]. Gene conversion events were tested using GENECONV version 1.81a [29], with 10,000 permutations. In order to avoid any mismatches within the gene conversion tracts, g-scale value was set to 0 [29, 30]. Significant gene conversion events were considered when the global *p* value was lower than 0.05 [30].

In order to assess phylogenetic relationships of MHCIIB and MHCIIY in galliform species, fifteen available MHCIIB sequences and four available MHCIIY sequences were downloaded from the GenBank (S2 Table). Phylogenetic trees were constructed following maximum likelihood (ML), which was performed on the RAxML Web server (http://phylobench.vital-it.ch/raxml-bb/) [31] with 100 rapid bootstrap replicates. The MHCIIB sequence of the Eurasian coot (*Fulica atra*) (GenBank accession no.: KF924779)[32] was used as outgroup. Furthermore, phylogenetic networks were constructed using the program SplitsTree4 [33] and employed the Neighbour-Net method [34] with Jukes-Cantor distances. Under complex models of evolution involving gene loss and duplication, hybridization, horizontal gene transfer or recombination, phylogenetic networks can provide a useful representation of the genetic relationships among sequences as compared to traditional phylogenetic trees [35].

## Results

### MHCIIB exon 2 sequence diversity

The primers successfully amplified the target fragment in all 73 individuals. Total 1046 clones derived from genomic DNA of 73 *S*. *humiae* (average 14 clones per individual) were sequenced and 24 distinct nucleotide variants of MHCIIB exon 2 were identified according to the criteria summarized by Kennedy et al. [22]. Because MHCIIB exon 2 are multilocus in galliform species and lack uniform gene names, for instance BLB for *Gallus gallus*, DAB for *Phasianus colchicus* [36], we simply name our alleles Syhu-DAB*01–24 (GenBank accession no.: KJ817332-KJ817355) according to the nomenclature of Klein et al. [37] regardless of phylogenetic analysis. All obtained *Syhu-DAB* sequences were of 222 bp length without stop codons and indels. The 222 bp nucleotide sequences exhibited high polymorphism with 84 variable nucleotide sites. Pairwise sequence divergence among alleles ranged from 0.5% to 30.1%, with a mean of 16.8%. Average nucleotide diversity was *π* = 0.14291, and the average number of nucleotide differences among alleles was *k* = 31.72. Amino acid alignment yielded 39 variable codons out of 74 total codons examined. The number of amino acid differences per site (*p* distance) ranged from 0 to 40.5%, with a mean of 23.1%. Following the three-dimensional structure of the human class II antigen [21], the 74 codon sequences were divided into ABS and non-ABS; the former contains 21 codons and the latter has 53 codons. Among ABS positions, 85.7% (18 out of 21) showed variation, and 39.6% (21 out of 53) showed variation in non-ABS positions. Nucleotide diversity (*π*) was 2.7 times higher at ABS (0.270) than the non-ABS (0.098).

Individuals displayed between one to seven variants. Of the 73 *S*. *humiae* individuals, 10 displayed one allele (13.7%), 24 displayed two alleles (32.9%), 28 displayed three alleles (38.3%), 6 displayed four alleles (8.2%), 4 displayed five alleles (5.5%), and only one displayed seven alleles (1.3%), with a mean of 2.6 (S1 Table). The most abundant allele, Syhu-DAB*01 was present in 57 individuals out of 73 (78%). The rarest allele, Syhu-DAB*23–24 was only detected in a single individual (*Syhu*32).

Seven *DAB* alleles (Syhu-DAB*01, Syhu-DAB*02, Syhu-DAB*03, Syhu-DAB*04, Syhu-DAB*05, Syhu-DAB*08 and Syhu-DAB*11) (Fig. 1B) were shared among four groups. Six rare alleles were detected at Group 1 (Syhu-DAB*22) and Group 3 (Syhu-DAB*16, Syhu-DAB*20, Syhu-DAB*21, Syhu-DAB*22, Syhu-DAB*23 and Syhu-DAB*24) (Fig. 1B).

### Balancing selection, recombination and gene conversion for the MHCIIB exon 2 region

Following the modified Nei-Gojobori/Jukes-Cantor [25] method, the ratios of *ω* (*d*_N_ ⁄ *d*_S_) were 0.921 in the overall MHCIIB exon 2 region, 1.109 in the ABS, and 0.803 in the non-ABS. Based on the Z-test of balancing selection, although these differences were not significant for all sites, ABS and non-ABS, non-synonymous substitutions per site for ABS sites outnumbered the synonymous substitutions per site, suggesting balancing selection at the ABS (Table 1).

**Table 1:** The estimated rates of non-synonymous (*d*_N_) and synonymous (*d*_S_) substitutions for antigen-binding (ABS) and non-antigen-binding (non-ABS) amino acid positions and their ratios (*ω*).

Positions	Codons	π	*d*_N_ (± SE)	*d*_S_ (± SE)	ω	p	Z (stat)
ABS	21	0.270	0.365 ± 0.093	0.329 ± 0.109	1.109	0.753 (ns)	0.315
non-ABS	53	0.098	0.102 ± 0.024	0.127 ± 0.043	0.803	0.568 (ns)	-0.572
All	74	0.147	0.165 ± 0.029	0.179 ± 0.041	0.921	0.752 (ns)	-0.317

*p*, the probability that *d*_N_ and *d*_S_ are different for Z-test with HA: *d*_N_ = / = *d*_S_; ns, not significant.

**Figure 2:**
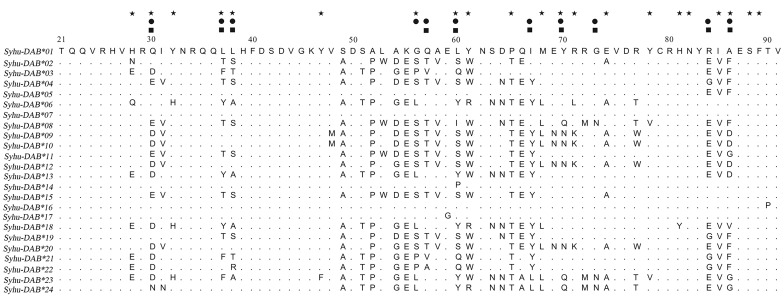
Alignment of the putative amino acid sequences of 24 MHCIIB exon 2 alleles in *Syrmaticus humiae*. Dots indicate identity with the top sequence (Syhu-DAB*01). Pentagrams (★) mark the antigen-binding sites (ABS) in the human MHC class II molecule [21]. Black squares (■, model M2a) and circles (●, model M8) mark sites under balancing selection based on CODEML analysis.

The maximum likelihood estimates of parameters under random-site models of variable *ω* across sites are listed in Table 2. Values of *ω* were significantly higher than one under M2a and M8 models that allow for balancing selection (*ω*_2_ = 4.945 in M2a and *ω* = 4.395 in M8). The likelihood ratio test of hypotheses showed statistically significant values (M2a vs. M1a: *df* = 2, 2△InL = 39.73, *p* < 0.001; M8 vs. M7: *df* = 2, 2△InL = 40.30, *p* < 0.001), revealing the balancing selection model is a better fit to the data compared to other models. Under M2a model, ten codons were identified as being under balancing selection (57th with *p* > 95% probability; 37th, 67th and 86th with *p* > 99% probability) (Table 2 and Fig. 2). Under M8 model, eleven codons were identified as being under balancing selection (30th, 38th, 57th, 60th and 84th with *p* > 95% probability; 37th, 57th, 67th and 86th with *p* > 99% probability) (Table 2 and Fig. 2). Seven out of eleven in M8 correspond to the ABS residues known from humans [21], and the other sites were adjacent to putative ABS (Fig. 2).

**Table 2:** Positively selected sites of Hume’s pheasant MHCIIB exon 2 sequences from implementing neutral (M1a, M7) and selection (M2a, M8) models in CODEML [26].

Model	lnL	Parameter estimates	Positively selected sites
M1a (Neutral)	-1266.512	*p*_0_ = 0.584, *p*_1_ = 0.416	Not allowed
M2a (Selection)	-1246.647	*p*_0_ = 0.539, *p*_1_ = 0.320, *p*_2_ = 0.139, *ω*_2_ = 4.945	30th, **37th**, 38th, *57th*, 60th, **67th**, 70th, 73rd, 84th, **86th**
M7 (β)	-1267.350	*p* = 0.116, *q* = 0.171	Not allowed
M8 (β & ω >1)	-1247.198	*p*_0_ = 0.855, *p*_1_ = 0.144, *p* = 0.108, *q* = 0.183, *ω* = 4.395	*30th*, **37th**, *38th*, 56th, **57th**, *60th*, **67th**, 70th, 73rd, *84th*, **86th**

Sites inferred under selection at the 99% level are listed in bold, and those inferred at the 95% level are shown in italics, underlined sites correspond to the ABS residues in humans. Number of sites is corresponding to that of HLA-DRB specified by Brown et al. [21].

The RDP, GENECONV and MaxChi methods of recombination detection found one, three and one recombination events respectively, indicating that recombination or gene conversion events have occurred at MHCIIB exon 2. Seventy-five significant gene conversion events were observed within the *S*. *humiae* MHCIIB locus (S3 Table). Conversion tracts varied in size between 24 to 119 bp. Two of the 24 MHCIIB exon 2 sequences described in this study shared no fragment with other sequences (*Syhu-DAB12* and *Syhu-DAB24*).

### Trans-species polymorphism

**Figure 3:**
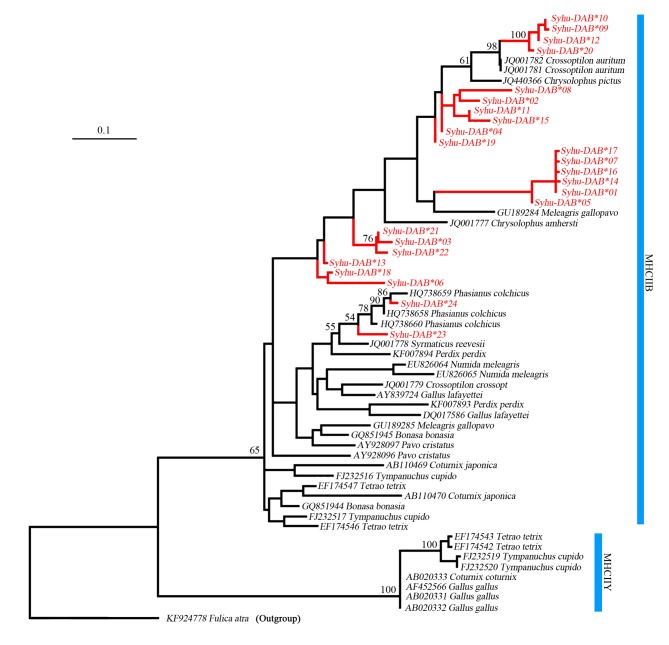
Maximum likelihood tree of exon 2 MHCIIB and MHCIIY sequences across galliform species. The sequence of DAB of the Eurasian coot (*Fulica atra*) (GenBank accession no.: KF924779)[32] was treated as an outgroup. Red color indicates MHCIIB exon 2 of *Syrmaticus humiae*. Only bootstrap values 50% were shown. For GenBank accession numbers and references see S2 Table.

The trans-species mode was detected according to phylogenetic trees. The MHCIIB sequences formed a clearly separated cluster from MHCIIY sequences, in agreement with Promerová et al. [30]. MHCIIB exon 2 of *S*. *humiae* were embedded within MHCIIB group (Fig. 3). *S*. *humiae* alleles, however, did not cluster together, but instead were scattered through the tree. The network relationships between different galliform exon 2 sequences that were isolated in different species of the same genus appeared to be more similar to each other than to sequences within species, suggesting the trans-species evolutionary model (S1 Fig.).

## Discussion

In this study, we have characterized MHCIIB exon 2 variation in a wild population of *S*. *humiae*. Following Kennedy’s et al. criteria [22], twenty-four distinct MHCIIB exon 2 alleles have been identified from 73 individuals, indicating that a relatively high level of MHCIIB variation was found in the *S*. *humiae* population. Among 73 individuals, most individuals have one to five alleles (72 out of 73). Following a strict cloning criterion, one individual (*Syhu*63) in our test possessed up to seven distinct variations. Due to just one individual having more than five alleles and the remaining individuals having less than five alleles, we suggested that there were at least three MHCIIB loci of *S*. *humiae* from this study. Similar results have been reported in some galliform species such as *Coturnix japonica* (7 loci), *Gallus gallus* (2 loci), *Meleagris gallopavo* (3 loci), *Pavo cristatus* (3 loci), *Phasianus colchicus* (3 loci), *Tympanuchus cupido* (4 loci), *Perdix perdix* (2 loci) [30, 36, 38–44]. Moreover, compared to some non-Passeriformes birds, *S*. *humiae* populations have higher allelic diversity. For instance, ten alleles were identified in the captive red jungle fowl (*Gallus gallus*) population of 80 individuals [45], twelve alleles were found in 108 individuals of the Grey Partridge (*Perdix perdix*) [30], two hundred and sixty-five alleles were found in 902 individuals of the Eurasian Coot (*Fulica atra*) [32], sixteen alleles were detected in 164 individuals of the black grouse (*Tetrao tetrix*) [46], and twenty-four alleles were detected in 40 individuals of the greater prairie-chicken (*Tympanuchus cupido*) [47]. Despite the wild *S*. *humiae* population declining in numerical abundance and geographic range, neutral markers (mitochondrial DNA, D-Loop) displayed relatively high genetic diversity and lacked striking genetic differentiation [8]. This may be for two reasons: (i) that the impact of fragmentation is still too recent to have had any effects detectable at the genetic level; and (ii) species-specific biological behavior. Fieldwork surveys suggested that *S*. *humiae* populations have suffered severe declines in the wild since the 1980s [5, 6]. However, political measures from 2000 onwards, such as legislation of the newly emended wild animal law and creation of national parks, have seen the trend towards habitat fragmentation slow. According to a recent survey, population size of *S*. *humiae* is growing [48]. Additionally, the dispersal of *S*. *humiae* is commonly assumed to be high [6] which can help buffer against the effects of severe habitat fragmentation [49]. Thus, neutral and selective markers have revealed high genetic diversity in this *S*. *humiae* population.

MHCIIB allele "Syhu-DAB*01" was the most frequent among individuals (57 out of 73, 78%). This suggests that Syhu-DAB*01 may have been favored by selection in the *S*. *humiae* population, probably because of its important role in the immune response against pathogens, adapting to environments or for demographic reasons. Among four geographical groups, we found seven shared alleles (Fig. 1B), indicating its central immune role in the *S*. *humiae* population. At the same time, we also observed six rare MHCIIB alleles (Fig. 1B). Although we cannot obtain enough samples from its ranges, it is probable that the *S*. *humiae* population has been separated by habitat fragmentation, so that some alleles are only distributed to some small populations, consistent with D-Loop results [8].

Balancing selection and gene conversion might have played an important role in the evolution of MHCIIB diversity in *S*. *humiae*. Whatever the maximum likelihood estimate or the modified Nei-Gojobori/Jukes-Cantor [25] method, the values of *ω* were higher than unity, confirming the influence of balancing selection on the MHCIIB locus of *S*. *humiae*. The likelihood ratio test of hypotheses (M1a vs. M2a and M7 vs. M8) showed statistically significant values (P < 0.001), suggesting a balancing selection model is a better fit to the data compared to other models. Thus, our results indicated that the MHCIIB exon 2 of *S*. *humiae* has been the target of balancing selection. Under the BEB analysis, ten sites (30th, 37th, 38th, 57th, 60th, 67th, 70th, 73rd, 84th, 86th) in M2a and eleven sites (30th, 37th, 38th, 56th, 57th, 60th, 67th, 70th, 73rd, 84th, 86th) in M8 have been confirmed to be subject to balancing selection with high *ω* values (Table 2). Among these sites, six sites (30th, 37th, 38th, 60th, 70th, 86th) corresponded to the peptide binding site of human MHC class II HLA-DR [21]. Variation at these peptide binding sites may be favored by host-pathogen co-evolution. In addition, gene conversion contributes significantly to sequence variations in *S*. *humiae*. All 11 sites in the putative peptide-binding region under balancing selection lay within conversion tracts (S3 Table). Especially given that these populations have undergone a bottleneck, gene conversion can be a crucial driver of allelic diversity [30], and create polymorphism much faster than point mutations by reshuffling polymorphism fragments among alleles thus leading to new haplotypes [50].

The phylogenetic tree and network indicated a pattern of MHCIIB trans-species evolution among galliform species, in agreement with the previous studies [30, 44]. The independent evolution of the BLB and YLB loci were supported clearly. The YLB in all species for which data were available formed a single cluster with 100% bootstrap support. *S*. *humiae* alleles did not form a monophyletic group, but were scattered through the tree instead. The galliform MHCIIB exon 2 sequences did not cluster by locus or by species. This may be caused by more frequent gene conversion among loci (concerted evolution) or by more recent gene duplication [18].

## Supporting Information

S1 Fig Neighbour-net constructed from exon 2 MHCIIB and MHCIIY sequences across galliform species using Jukes-Cantor distances. (TIF)

S1 Table Sampling localities and allele distributions. (DOC)

S2 Table Fifteen taxa MHCIIB and four taxa MHCIIY sequences of Galliformes were used in the phylogenetic analysis, with references and GenBank accession numbers. (DOC)

S3 Table Gene conversion events among 24 nucleotide sequences of MHCIIB exon 2 in *Syrmaticus humiae*. (DOC)
