# Supplementary figures and images for: Genetic Variation of the Major Histocompatibility Complex (MHC Class II B Gene) in the Threatened Hume’s Pheasant, Syrmaticus humiae
Source: PLoS One. 2015 Jan 28;10(1):e0116499. doi: 10.1371/journal.pone.0116499 (PMC4309451; doi:10.1371/journal.pone.0116499)

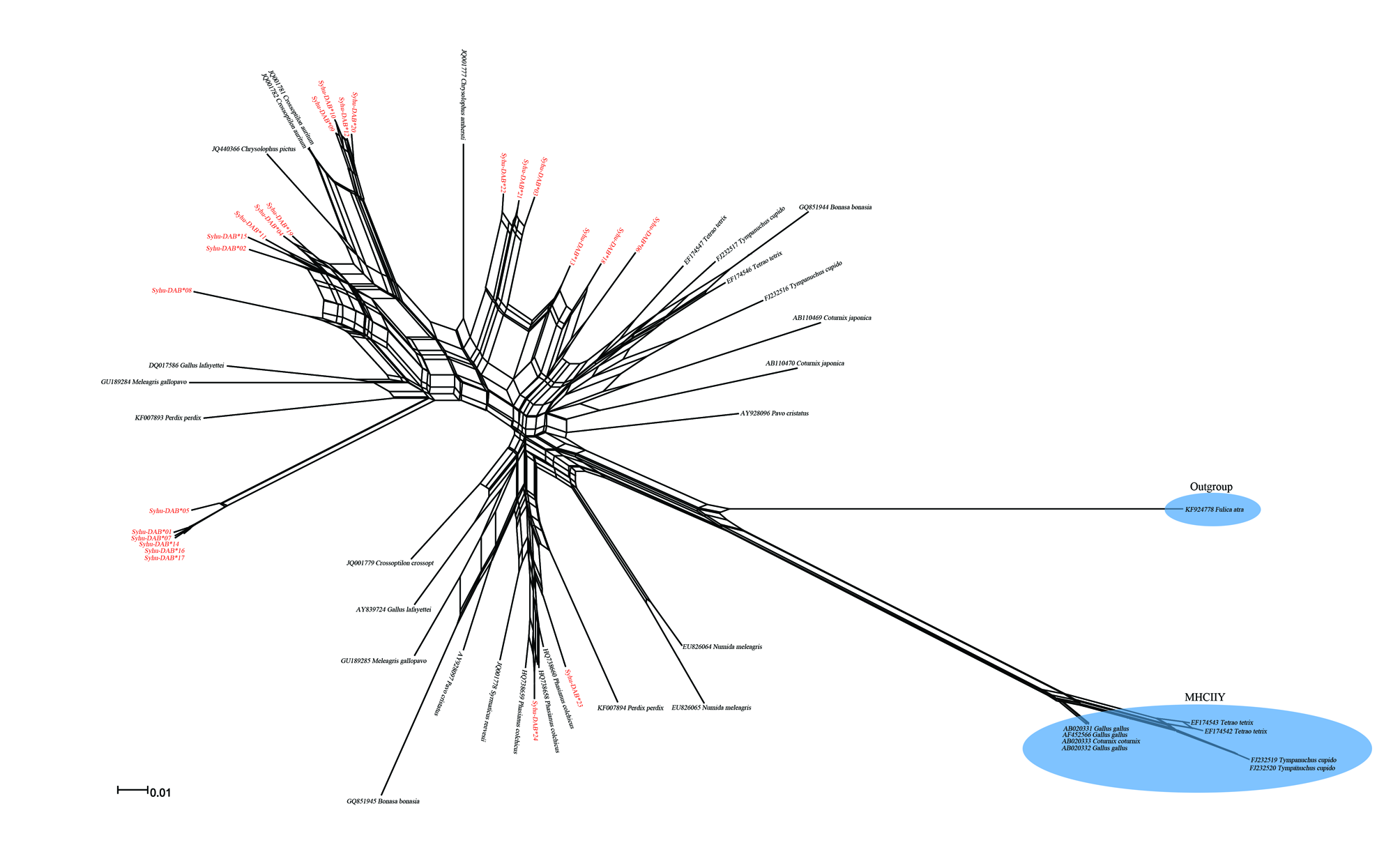

Supplement: S1 Fig — (TIF) [file pone.0116499.s001.tif]
