# Supplementary material for: Genetic Variation of the Major Histocompatibility Complex (MHC Class II B Gene) in the Threatened Hume’s Pheasant, Syrmaticus humiae
Source: PLoS One. 2015 Jan 28;10(1):e0116499. doi: 10.1371/journal.pone.0116499 (PMC4309451; doi:10.1371/journal.pone.0116499)
Supplement: S1 Table — (DOC) [file pone.0116499.s002.doc]

**Supporting Information**

**Table S1. Sampling localities and allele distributions.**

| ID | Archived No. | Sampling locality | Alleles | | | | | | | | | | | | | | | | | | | | | | | |
| --- | --- | --- | --- | --- | --- | --- | --- | --- | --- | --- | --- | --- | --- | --- | --- | --- | --- | --- | --- | --- | --- | --- | --- | --- | --- | --- |
|  |  |  | 1 | 2 | 3 | 4 | 5 | 6 | 7 | 8 | 9 | 10 | 11 | 12 | 13 | 14 | 15 | 16 | 17 | 18 | 19 | 20 | 21 | 22 | 23 | 24 |
| 1 | syhu8 | Jinzhongshan National Nature  Reserve, Longlin County | 1 |  | 1 |  |  |  |  |  |  |  |  |  |  |  |  |  |  | 1 |  |  |  |  |  |  |
| 2 | syhu80 | Longhuo village, Longlin County | 1 |  |  | 1 |  | 1 |  |  |  |  |  |  |  |  |  |  |  |  |  |  |  |  |  |  |
| 3 | syhu83 | Longhuo village, Longlin County | 1 |  |  |  |  | 1 |  |  |  |  |  |  |  |  |  |  |  |  |  |  |  |  |  |  |
| 4 | syhu10 | Guzhang Forestry Center, Xilin County | 1 |  |  |  |  |  |  |  |  |  |  |  |  |  |  |  |  |  |  |  |  |  |  |  |
| 5 | syhu11 | Guzhang Forestry Center, Xilin County | 1 |  |  |  |  |  |  |  |  |  |  |  |  |  |  |  |  |  |  |  |  |  |  |  |
| 6 | syhu54 | Fuda village, Tianlin County |  |  |  |  | 1 |  |  |  |  |  |  |  |  |  |  |  |  |  |  |  |  | 1 |  |  |
| 7 | syhu55 | Fuda village, Tianlin County | 1 |  |  |  | 1 |  |  | 1 |  |  |  |  |  |  |  |  |  |  |  |  |  |  |  |  |
| 8 | syhu58 | Fuda village, Tianlin County | 1 |  |  |  | 1 | 1 |  | 1 |  |  |  |  |  |  |  |  |  |  |  |  |  |  |  |  |
| 9 | syhu59 | Fuda village, Tianlin County | 1 |  | 1 |  |  |  |  |  |  |  |  |  |  |  |  |  |  |  |  |  |  |  |  |  |
| 10 | syhu60 | Fuda village, Tianlin County | 1 |  | 1 |  |  |  |  |  |  |  |  |  |  |  |  |  |  |  |  |  |  |  |  |  |
| 11 | syhu61 | Fuda village, Tianlin County | 1 |  | 1 |  |  |  |  |  |  |  |  |  |  |  |  |  |  |  |  |  |  |  |  |  |
| 12 | syhu62 | Fuda village, Tianlin County |  |  | 1 |  |  |  |  |  |  |  |  |  |  |  |  |  |  |  |  |  |  |  |  |  |
| 13 | syhu63 | Fuda village, Tianlin County | 1 | 1 |  | 1 |  | 1 |  |  | 1 |  | 1 |  |  |  |  |  |  |  |  |  |  | 1 |  |  |
| 14 | syhu64 | Fuda village, Tianlin County | 1 |  |  |  |  |  |  |  |  |  |  |  |  |  |  |  |  |  |  |  |  |  |  |  |
| 15 | syhu65 | Fuda village, Tianlin County | 1 |  |  |  |  |  |  |  |  |  |  |  |  |  |  |  |  |  |  |  |  |  |  |  |
| 16 | syhu66 | Fuda village, Tianlin County | 1 |  |  |  |  |  |  |  |  |  |  |  | 1 |  |  |  |  |  |  |  |  |  |  |  |
| 17 | syhu67 | Fuda village, Tianlin County |  |  | 1 |  |  |  |  |  |  |  |  |  |  |  |  |  |  |  |  |  |  |  |  |  |
| 18 | syhu127 | Fuda village, Tianlin County | 1 | 1 |  |  |  |  |  |  | 1 |  |  |  |  |  |  |  |  |  |  |  |  |  |  |  |
| 19 | syhu128 | Fuda village, Tianlin County | 1 | 1 |  |  |  |  |  |  | 1 |  |  |  |  |  |  |  |  |  |  |  |  |  |  |  |
| 20 | syhu129 | Fuda village, Tianlin County | 1 | 1 |  |  |  |  |  |  | 1 |  |  |  |  |  |  |  |  |  |  |  |  |  |  |  |
| 21 | syhu130 | Fuda village, Tianlin County | 1 | 1 |  |  |  | 1 |  |  | 1 |  |  |  |  |  | 1 |  |  |  |  |  |  |  |  |  |
| 22 | syhu131 | Fuda village, Tianlin County | 1 | 1 |  |  |  |  |  |  | 1 |  |  |  |  |  |  |  |  |  |  |  |  |  |  |  |
| 23 | syhu132 | Fuda village, Tianlin County | 1 |  |  |  |  |  | 1 |  |  |  |  |  |  |  |  |  |  |  |  |  |  |  |  |  |
| 24 | syhu133 | Fuda village, Tianlin County | 1 | 1 |  |  |  |  | 1 |  |  |  |  |  |  |  |  |  |  |  |  |  |  |  |  |  |
| 25 | syhu134 | Fuda village, Tianlin County | 1 |  |  |  |  |  | 1 |  |  |  |  |  |  |  |  |  |  |  |  |  |  |  |  |  |
| 26 | syhu135 | Fuda village, Tianlin County | 1 |  |  |  |  | 1 | 1 |  |  |  |  |  |  |  |  |  |  |  |  |  |  |  |  |  |
| 27 | syhu136 | Fuda village, Tianlin County | 1 |  |  |  |  |  | 1 |  |  |  |  |  |  | 1 |  |  |  |  |  |  |  |  |  |  |
| 28 | syhu25 | Luoxi village, Leye County |  | 1 | 1 | 1 |  |  |  |  |  |  |  |  |  |  |  |  |  |  |  |  |  |  |  |  |
| 29 | syhu26 | Luoxi village, Leye County | 1 |  |  |  |  | 1 |  |  |  |  |  |  |  |  |  |  | 1 |  |  |  |  |  |  |  |
| 30 | syhu27 | Luoxi village, Leye County | 1 |  |  |  |  |  | 1 |  |  |  |  |  |  |  |  |  |  |  |  |  |  |  |  |  |
| 31 | syhu68 | Luoxi village, Leye County |  |  | 1 |  |  |  |  |  |  |  |  |  |  |  |  |  |  |  |  |  |  |  |  |  |
| 32 | syhu69 | Luoxi village, Leye County | 1 |  |  | 1 |  |  |  |  |  | 1 |  |  |  | 1 | 1 |  |  |  |  |  |  |  |  |  |
| 33 | syhu70 | Luoxi village, Leye County | 1 |  |  |  |  |  |  | 1 |  |  | 1 |  |  |  |  |  |  |  |  |  |  |  |  |  |
| 34 | syhu71 | Luoxi village, Leye County | 1 |  | 1 |  |  |  |  |  |  |  |  |  |  |  |  |  |  |  |  |  |  |  |  |  |
| 35 | syhu74 | Luoxi village, Leye County |  | 1 |  | 1 | 1 |  |  |  |  |  |  |  |  |  |  |  |  |  |  |  |  |  |  |  |
| 36 | syhu75 | Luoxi village, Leye County |  | 1 |  | 1 | 1 |  |  |  |  |  |  |  |  |  |  |  |  |  | 1 |  |  |  |  |  |
| 37 | syhu109 | Luoxi village, Leye County | 1 |  |  |  |  |  |  |  |  |  |  |  |  | 1 |  |  |  |  |  |  |  |  |  |  |
| 38 | syhu110 | Luoxi village, Leye County |  |  | 1 |  |  |  |  |  |  |  | 1 |  |  |  |  |  |  |  |  |  |  |  |  |  |
| 39 | syhu114 | Luoxi village, Leye County | 1 |  |  | 1 |  |  |  |  |  |  |  |  |  |  |  |  |  |  |  |  |  |  |  |  |
| 40 | syhu116 | Luoxi village, Leye County | 1 |  |  |  |  |  |  |  |  |  |  | 1 |  |  |  |  |  |  |  |  |  |  |  |  |
| 41 | syhu117 | Xialao village, Tian'e County | 1 |  |  |  | 1 |  |  |  |  |  |  |  |  |  |  |  |  |  |  |  |  |  |  |  |
| 42 | syhu119 | Xialao village, Tian'e County | 1 |  |  |  |  | 1 |  |  |  |  |  |  |  |  |  |  | 1 |  |  |  |  |  |  |  |
| 43 | syhu123 | Xialao village, Tian'e County | 1 |  |  |  |  |  |  |  |  | 1 |  |  | 1 |  | 1 |  |  |  |  |  |  |  |  |  |
| 44 | syhu16 | Pojie village, Tian'e County | 1 |  |  |  | 1 |  |  | 1 |  |  |  |  |  |  |  |  |  |  |  |  |  |  |  |  |
| 45 | syhu18 | Pojie village, Tian'e County | 1 |  |  |  |  |  |  |  |  |  |  | 1 |  |  |  |  |  |  |  |  |  |  |  |  |
| 46 | syhu19 | Pojie village, Tian'e County |  | 1 |  | 1 |  |  | 1 |  |  |  |  |  |  |  |  |  |  |  |  |  |  |  |  |  |
| 47 | syhu21 | Pojie village, Tian'e County | 1 |  |  |  |  |  |  |  |  |  |  |  |  |  |  |  |  |  |  |  |  |  |  |  |
| 48 | syhu32 | Pojie village, Tian'e County |  |  |  |  |  |  |  |  |  |  |  |  |  |  |  |  |  |  |  |  |  |  | 1 | 1 |
| 49 | syhu38 | Pojie village, Tian'e County | 1 |  |  | 1 |  |  |  |  |  |  |  |  |  |  |  |  |  |  | 1 |  |  |  |  |  |
| 50 | syhu39 | Pojie village, Tian'e County | 1 | 1 |  | 1 |  |  |  |  |  |  |  |  |  |  |  | 1 |  |  |  |  |  |  |  |  |
| 51 | syhu41 | Pojie village, Tian'e County | 1 |  |  |  | 1 |  |  |  |  |  |  |  |  |  |  |  |  |  |  |  |  |  |  |  |
| 52 | syhu42 | Pojie village, Tian'e County | 1 |  |  |  |  |  |  |  |  |  |  |  |  |  |  |  |  |  |  | 1 |  |  |  |  |
| 53 | syhu46 | Pojie village, Tian'e County | 1 |  |  |  |  |  |  |  |  |  |  |  |  |  |  | 1 |  |  |  | 1 |  |  |  |  |
| 54 | syhu48 | Pojie village, Tian'e County | 1 |  |  |  |  |  |  |  |  |  |  |  |  |  |  |  |  |  |  |  | 1 |  |  |  |
| 55 | syhu78 | Pojie village, Tian'e County | 1 |  |  |  | 1 |  |  |  |  |  |  |  |  |  |  |  | 1 |  |  |  |  |  |  |  |
| 56 | syhu97 | Pojie village, Tian'e County |  |  |  |  |  |  |  |  |  | 1 |  | 1 |  |  |  | 1 |  |  |  |  | 1 |  |  |  |
| 57 | syhu98 | Pojie village, Tian'e County | 1 |  |  |  |  | 1 |  |  |  | 1 |  |  |  |  | 1 |  |  |  |  |  |  |  |  |  |
| 58 | syhu99 | Pojie village, Tian'e County | 1 | 1 |  |  |  | 1 |  |  | 1 | 1 |  |  |  |  |  |  |  |  |  |  |  |  |  |  |
| 59 | syhu101 | Pojie village, Tian'e County | 1 |  | 1 |  |  |  |  |  |  | 1 |  |  |  |  |  |  |  |  |  |  |  |  |  |  |
| 60 | syhu104 | Pojie village, Tian'e County |  |  | 1 |  |  |  |  |  |  |  |  | 1 |  |  |  |  |  | 1 |  |  |  |  |  |  |
| 61 | syhu105 | Pojie village, Tian'e County |  |  |  |  | 1 |  |  | 1 |  |  |  |  |  |  |  |  |  |  |  |  |  |  |  |  |
| 62 | syhu106 | Pojie village, Tian'e County | 1 |  |  |  |  |  |  |  |  | 1 |  |  | 1 |  |  |  |  |  |  |  |  |  |  |  |
| 63 | syhu107 | Pojie village, Tian'e County | 1 |  |  |  |  |  |  |  |  |  |  |  | 1 |  |  |  |  |  |  |  |  |  |  |  |
| 64 | syhu108 | Pojie village, Tian'e County | 1 |  |  |  |  |  |  |  |  |  | 1 |  |  |  |  |  |  |  |  |  |  |  |  |  |
| 65 | syhu53 | Luodian County |  |  | 1 |  |  |  |  |  |  |  |  |  |  |  |  |  |  |  |  |  |  |  |  |  |
| 66 | syhu86 | Luodian County | 1 | 1 |  | 1 |  |  |  |  |  |  |  |  |  |  |  |  |  |  |  |  |  |  |  |  |
| 67 | syhu87 | Luodian County | 1 |  | 1 | 1 |  |  |  |  |  |  |  |  |  |  |  |  |  |  |  |  |  |  |  |  |
| 68 | syhu88 | Luodian County |  | 1 | 1 | 1 |  |  |  |  |  |  |  |  |  |  |  |  |  |  |  |  |  |  |  |  |
| 69 | syhu90 | Luodian County | 1 |  |  |  |  |  |  |  |  |  |  |  |  |  |  |  |  |  |  |  |  |  |  |  |
| 70 | syhu93 | Luodian County | 1 |  |  |  | 1 |  |  | 1 |  |  |  |  |  |  |  |  |  |  |  |  |  |  |  |  |
| 71 | syhu96 | Luodian County | 1 |  | 1 |  | 1 |  |  | 1 |  |  |  |  |  | 1 |  |  |  |  |  |  |  |  |  |  |
| 72 | syhu112 | Luodian County |  |  |  |  | 1 |  |  |  |  |  | 1 |  |  |  |  |  |  |  |  |  |  |  |  |  |
| 73 | syhu113 | Luodian County | 1 |  |  |  |  |  |  |  |  |  | 1 | 1 |  |  |  |  |  |  |  |  |  |  |  |  |
