# Supplementary material for: Genetic Variation of the Major Histocompatibility Complex (MHC Class II B Gene) in the Threatened Hume’s Pheasant, Syrmaticus humiae
Source: PLoS One. 2015 Jan 28;10(1):e0116499. doi: 10.1371/journal.pone.0116499 (PMC4309451; doi:10.1371/journal.pone.0116499)
Supplement: S2 Table — (DOC) [file pone.0116499.s003.doc]

**Supporting Information**

**Table S2. Fifteen taxa MHCIIB and four taxa MHCIIY sequences of Galliformes were used in the phylogenetic analysis, with references and GenBank accession numbers.**

| ID | Species | Abbreviation | English name | GenBank Accession no. | Ref. |
| --- | --- | --- | --- | --- | --- |
| BLB sequences | | | | | |
| 1 | *Chrysolophus amherstiae* | Cham | Lady Amherst's Pheasant | JQ001777 | 1 |
| 2 | *Syrmaticus reevesii* | Syre | Reeves's Pheasant | JQ001778 | 1 |
| 3 | *Crossoptilon crossoptilon* | Crcr | White Eared Pheasant | JQ001779 | 1 |
| 4 | *Crossoptilon auritum* | Crau | Blue Eared Pheasant | JQ001781 | 1 |
| 5 | *Crossoptilon auritum* | Crau | Blue Eared Pheasant | JQ001782 | 1 |
| 6 | *Bonasa bonasia* | Bobo | Hazel Grouse | GQ851944 | 2 |
| 7 | *Bonasa bonasia* | Bobo | Hazel Grouse | GQ851945 | 2 |
| 8 | *Gallus lafayettei* | Gala | Ceylon jungle fowl | AY839724 | 3 |
| 9 | *Gallus lafayettei* | Gala | Ceylon jungle fowl | DQ017586 | 3 |
| 10 | *Numida meleagris* | Nume | Helmeted Guineafowl | EU826065 | 4 |
| 11 | *Numida meleagris* | Nume | Helmeted Guineafowl | EU826064 | 4 |
| 12 | *Pavo cristatus* | Pacr | Indian Peafowl | AY928096 | 5 |
| 13 | *Pavo cristatus* | Pacr | Indian Peafowl | AY928097 | 5 |
| 14 | *Meleagris gallopavo* | Mega | Wild Turkey | GU189285 | 6 |
| 15 | *Meleagris gallopavo* | Mega | Wild Turkey | GU189284 | 6 |
| 16 | *Tympanuchus cupido* | Tycu | Greater Prairie Chicken | FJ232517 | 7 |
| 17 | *Tympanuchus cupido* | Tycu | Greater Prairie Chicken | FJ232516 | 7 |
| 18 | *Tetrao tetrix* | Tete | Black Grouse | EF174546 | 8 |
| 19 | *Tetrao tetrix* | Tete | Black Grouse | EF174547 | 8 |
| 20 | *Phasianus colchicus* | Phco | Common Pheasant | HQ738659 | 9 |
| 21 | *Phasianus colchicus* | Phco | Common Pheasant | HQ738660 | 9 |
| 22 | *Phasianus colchicus* | Phco | Common Pheasant | HQ738658 | 9 |
| 23 | *Coturnix japonica* | Coja | Japanese Quail | AB181870 | 10 |
| 24 | *Coturnix japonica* | Coja | Japanese Quail | AB110469 | 11 |
| 25 | *Chrysolophus pictus* | Chpi | Golden Pheasant | JQ440366 | 12 |
| 26 | *Perdix perdix* | Pepe | Grey Partridge | KF007893 | 13 |
| 27 | *Perdix perdix* | Pepe | Grey Partridge | KF007894 | 13 |
| 28 | *Fulica atra* | Fuat | Eurasian coot | KF924779 | 14 |
| YLB sequences | | | | | |
| 29 | *Gallus gallus* | Gaga | Chicken | AB020331 | 15 |
| 30 | *Gallus gallus* | Gaga | Chicken | AB020332 | 15 |
| 31 | *Coturnix coturnix* | Coco | Common Quail | AB020333 | 15 |
| 32 | *Gallus gallus* | Gaga | Chicken | AF452566 | 16 |
| 33 | *Tetrao tetrix* | Tete | Black Grouse | EF174542 | 8 |
| 34 | *Tetrao tetrix* | Tete | Black Grouse | EF174543 | 8 |
| 35 | *Tympanuchus cupido* | Tycu | Greater Prairie Chicken | FJ232519 | 7 |
| 36 | *Tympanuchus cupido* | Tycu | Greater Prairie Chicken | FJ232520 | 7 |

**References**

1. Zhang Y, Zou F (2011) Cloning and Sequence Analysis of the MHC B-LB Gene in Five Pheasants. (http://www.ncbi.nlm.nih.gov/nuccore/JQ001777)

2. Strand TM, Höglund J. (2011) Genotyping of black grouse MHC class II B using reference Strand-Mediated Conformational Analysis (RSCA). BMC research notes 4(1): 183.

3. Lambourne MD, Si W, Niemiec PK, Read LR, Kariyawasam S, et al. (2005) Identification of novel polymorphisms in the B-LB locus of Gallus lafayettei. Anim Genet 36(5): 445-448

4. Kumar S, Mehra S, Shukla S, Mehra M, Gupta A, et al. (2008) Genetic polymorphism in BLB2 gene between guinea fowl and other poultry species (http://www.ncbi.nlm.nih.gov/nuccore/EU826065)

5. Hale ML, Verduijn MH, Moller AP, Wolff K, Petrie M (2009) Is the peacock's train an honest signal of genetic quality at the major histocompatibility complex? J Evol Biol 22: 1284-1294

6. Chaves LD, Faile GM, Krueth SB, Hendrickson JA, Reed KM (2010) Haplotype variation, recombination, and gene conversion within the turkey MHC-B locus. Immunogenetics 62(7): 465-477

7. Eimes JA, Bollmer JL, Dunn PO, Whittingham LA, Wimpee C (2010) Mhc class II diversity and balancing selection in greater prairie-chickens. Genetica 138(2): 265-271

8. Strand T, Westerdahl H, Hoglund J, V Alatalo R, Siitari H (2007) The Mhc class II of the Black grouse (*Tetrao tetrix*) consists of low numbers of B and Y genes with variable diversity and expression. Immunogenetics 59(9): 725-734

9. Baratti M, DESSÌ-FULGHERI F, Ambrosini R, BONISOLI-ALQUATI A, Caprioli M, et al. (2012). MHC genotype predicts mate choice in the ring-necked pheasant *Phasianus colchicus*. J Evol Biol 25(8): 1531-1542

10. Hosomichi K, Shiina T, Suzuki S, Tanaka M, Shimizu S, et al. (2006) The major histocompatibility complex (Mhc) class IIB region has greater genomic structural flexibility and diversity in the quail than the chicken. BMC Genomics 7: 322

11. Shimizu S, Shiina T, Hosomichi K, Takahashi S, Koyama T, et al. (2004) MHC class IIB gene sequences and expression in quails (*Coturnix japonica*) selected for high and low antibody responses. Immunogenetics 56(4): 280-291

12. Ye Q, He K, Wu SY, Wan QH (2012) Isolation of a 97-kb minimal essential MHC B locus from a new reverse-4D BAC library of the golden pheasant. PloS one 7:e32154

13. Promerová M, Králová T, Bryjová A, Albrecht T, Bryja J (2013) MHC Class IIB Exon 2 Polymorphism in the Grey Partridge (*Perdix perdix*) Is Shaped by Selection, Recombination and Gene Conversion. PloS one 8:e69135

14. Alcaide M, Muñoz J, Martínez-de la Puente J, Soriguer R, Figuerola J (2014) Extraordinary MHC class II B diversity in a non-passerine, wild bird: the Eurasian Coot *Fulica atra* (Aves: Rallidae). Ecol Evol 4:688-698

15. Nishibori M, Tsudzuki M, Yamamoto Y (1998) Characterization of pseudogenes designated Y-Lb III in chicken MHC class II (B-L) genes. (http://www.ncbi.nlm.nih.gov/nuccore/AB020331)

16. Soria LA, Iglesias GM, Jar AM, Miquel MC, Lopez OJ (2001) Genetic polymorphism in the MHC B-L locus of Camperos, a mixed breed of broilers. (http://www.ncbi.nlm.nih.gov/nuccore/AF452566)
