# Supplementary material for: Genetic Variation of the Major Histocompatibility Complex (MHC Class II B Gene) in the Threatened Hume’s Pheasant, Syrmaticus humiae
Source: PLoS One. 2015 Jan 28;10(1):e0116499. doi: 10.1371/journal.pone.0116499 (PMC4309451; doi:10.1371/journal.pone.0116499)
Supplement: S3 Table — (DOC) [file pone.0116499.s004.doc]

**Supporting Information**

**Table S3. Gene conversion events among 24 nucleotide sequences of MHCIIB exon 2 in Syrmaticus *humiae***

| Tract No. | Seq 1 | Seq 2 | Simulated *p* value | Start | End | Length | No. of polymorphisms | Total differences |
| --- | --- | --- | --- | --- | --- | --- | --- | --- |
| 1 | *Syhu-DAB*08* | *Syhu-DAB*15* | 0.0000 | 1 | 118 | 118 | 43 | 25 |
|  | *Syhu-DAB*08* | *Syhu-DAB*11* | 0.0010 | 1 | 118 | 118 | 43 | 20 |
| 2 | *Syhu-DAB*06* | *Syhu-DAB*18* | 0.0009 | 31 | 149 | 119 | 55 | 16 |
| 3 | *Syhu-DAB*09* | *Syhu-DAB*16* | 0.0451 | 32 | 81 | 50 | 13 | 44 |
|  | *Syhu-DAB*09* | *Syhu-DAB*17* | 0.0451 | 32 | 81 | 50 | 13 | 44 |
| 4 | *Syhu-DAB*02* | *Syhu-DAB*08* | 0.0270 | 32 | 118 | 87 | 36 | 19 |
|  | *Syhu-DAB*06* | *Syhu-DAB*13* | 0.0491 | 35 | 116 | 82 | 32 | 20 |
| 5 | *Syhu-DAB*13* | *Syhu-DAB*23* | 0.0169 | 82 | 136 | 55 | 32 | 22 |
| 6 | *Syhu-DAB*02* | *Syhu-DAB*10* | 0.0321 | 99 | 138 | 40 | 24 | 27 |
|  | *Syhu-DAB*10* | *Syhu-DAB*15* | 0.0421 | 99 | 141 | 43 | 27 | 24 |
|  | *Syhu-DAB*15* | *Syhu-DAB*20* | 0.0224 | 102 | 141 | 40 | 26 | 26 |
|  | *Syhu-DAB*11* | *Syhu-DAB*20* | 0.0393 | 102 | 141 | 40 | 26 | 25 |
| 7 | *Syhu-DAB*03* | *Syhu-DAB*07* | 0.0000 | 124 | 189 | 66 | 28 | 40 |
|  | *Syhu-DAB*03* | *Syhu-DAB*16* | 0.0000 | 124 | 189 | 66 | 28 | 40 |
|  | *Syhu-DAB*03* | *Syhu-DAB*17* | 0.0000 | 124 | 189 | 66 | 28 | 40 |
|  | *Syhu-DAB*01* | *Syhu-DAB*03* | 0.0000 | 124 | 189 | 66 | 28 | 39 |
|  | *Syhu-DAB*03* | *Syhu-DAB*14* | 0.0000 | 124 | 189 | 66 | 28 | 39 |
|  | *Syhu-DAB*03* | *Syhu-DAB*05* | 0.0000 | 124 | 222 | 99 | 36 | 32 |
|  | *Syhu-DAB*04* | *Syhu-DAB*13* | 0.0083 | 129 | 190 | 62 | 28 | 26 |
|  | *Syhu-DAB*13* | *Syhu-DAB*19* | 0.0083 | 129 | 190 | 62 | 28 | 26 |
|  | *Syhu-DAB*11* | *Syhu-DAB*13* | 0.0010 | 131 | 196 | 66 | 31 | 27 |
| 8 | *Syhu-DAB*02* | *Syhu-DAB*07* | 0.0190 | 137 | 160 | 24 | 17 | 38 |
|  | *Syhu-DAB*02* | *Syhu-DAB*16* | 0.0190 | 137 | 160 | 24 | 17 | 38 |
|  | *Syhu-DAB*02* | *Syhu-DAB*17* | 0.0190 | 137 | 160 | 24 | 17 | 38 |
|  | *Syhu-DAB*01* | *Syhu-DAB*02* | 0.0277 | 137 | 160 | 24 | 17 | 37 |
|  | *Syhu-DAB*02* | *Syhu-DAB*14* | 0.0277 | 137 | 160 | 24 | 17 | 37 |
| 9 | *Syhu-DAB*11* | *Syhu-DAB*21* | 0.0039 | 137 | 190 | 54 | 25 | 30 |
|  | *Syhu-DAB*11* | *Syhu-DAB*22* | 0.0077 | 137 | 190 | 54 | 25 | 29 |
|  | *Syhu-DAB*04* | *Syhu-DAB*22* | 0.0421 | 137 | 194 | 58 | 27 | 24 |
|  | *Syhu-DAB*19* | *Syhu-DAB*22* | 0.0421 | 137 | 194 | 58 | 27 | 24 |
| 10 | *Syhu-DAB*04* | *Syhu-DAB*21* | 0.0044 | 137 | 222 | 86 | 32 | 24 |
|  | *Syhu-DAB*19* | *Syhu-DAB*21* | 0.0044 | 137 | 222 | 86 | 32 | 24 |
| 11 | *Syhu-DAB*07* | *Syhu-DAB*13* | 0.0000 | 141 | 189 | 49 | 21 | 42 |
|  | *Syhu-DAB*07* | *Syhu-DAB*21* | 0.0000 | 141 | 189 | 49 | 21 | 42 |
|  | *Syhu-DAB*13* | *Syhu-DAB*16* | 0.0000 | 141 | 189 | 49 | 21 | 42 |
|  | *Syhu-DAB*13* | *Syhu-DAB*17* | 0.0000 | 141 | 189 | 49 | 21 | 42 |
|  | *Syhu-DAB*16* | *Syhu-DAB*21* | 0.0000 | 141 | 189 | 49 | 21 | 42 |
|  | *Syhu-DAB*17* | *Syhu-DAB*21* | 0.0000 | 141 | 189 | 49 | 21 | 42 |
|  | *Syhu-DAB*01* | *Syhu-DAB*13* | 0.0000 | 141 | 189 | 49 | 21 | 41 |
|  | *Syhu-DAB*01* | *Syhu-DAB*21* | 0.0000 | 141 | 189 | 49 | 21 | 41 |
|  | *Syhu-DAB*07* | *Syhu-DAB*22* | 0.0000 | 141 | 189 | 49 | 21 | 41 |
|  | *Syhu-DAB*13* | *Syhu-DAB*14* | 0.0000 | 141 | 189 | 49 | 21 | 41 |
|  | *Syhu-DAB*14* | *Syhu-DAB*21* | 0.0000 | 141 | 189 | 49 | 21 | 41 |
|  | *Syhu-DAB*16* | *Syhu-DAB*22* | 0.0000 | 141 | 189 | 49 | 21 | 41 |
|  | *Syhu-DAB*17* | *Syhu-DAB*22* | 0.0000 | 141 | 189 | 49 | 21 | 41 |
|  | *Syhu-DAB*01* | *Syhu-DAB*22* | 0.0004 | 141 | 189 | 49 | 21 | 40 |
|  | *Syhu-DAB*14* | *Syhu-DAB*22* | 0.0004 | 141 | 189 | 49 | 21 | 40 |
|  | *Syhu-DAB*04* | *Syhu-DAB*07* | 0.0006 | 141 | 189 | 49 | 21 | 39 |
|  | *Syhu-DAB*04* | *Syhu-DAB*16* | 0.0006 | 141 | 189 | 49 | 21 | 39 |
|  | *Syhu-DAB*04* | *Syhu-DAB*17* | 0.0006 | 141 | 189 | 49 | 21 | 39 |
|  | *Syhu-DAB*01* | *Syhu-DAB*04* | 0.0009 | 141 | 189 | 49 | 21 | 38 |
|  | *Syhu-DAB*04* | *Syhu-DAB*14* | 0.0009 | 141 | 189 | 49 | 21 | 38 |
|  | *Syhu-DAB*07* | *Syhu-DAB*19* | 0.0012 | 141 | 189 | 49 | 21 | 37 |
|  | *Syhu-DAB*16* | *Syhu-DAB*19* | 0.0012 | 141 | 189 | 49 | 21 | 37 |
|  | *Syhu-DAB*17* | *Syhu-DAB*19* | 0.0012 | 141 | 189 | 49 | 21 | 37 |
|  | *Syhu-DAB*01* | *Syhu-DAB*19* | 0.0019 | 141 | 189 | 49 | 21 | 36 |
|  | *Syhu-DAB*07* | *Syhu-DAB*11* | 0.0019 | 141 | 189 | 49 | 21 | 36 |
|  | *Syhu-DAB*11* | *Syhu-DAB*16* | 0.0019 | 141 | 189 | 49 | 21 | 36 |
|  | *Syhu-DAB*11* | *Syhu-DAB*17* | 0.0019 | 141 | 189 | 49 | 21 | 36 |
|  | *Syhu-DAB*14* | *Syhu-DAB*19* | 0.0019 | 141 | 189 | 49 | 21 | 36 |
|  | *Syhu-DAB*01* | *Syhu-DAB*11* | 0.0028 | 141 | 189 | 49 | 21 | 35 |
|  | *Syhu-DAB*11* | *Syhu-DAB*14* | 0.0028 | 141 | 189 | 49 | 21 | 35 |
|  | *Syhu-DAB*05* | *Syhu-DAB*21* | 0.0010 | 141 | 190 | 50 | 22 | 36 |
|  | *Syhu-DAB*05* | *Syhu-DAB*22* | 0.0010 | 141 | 190 | 50 | 22 | 36 |
|  | *Syhu-DAB*04* | *Syhu-DAB*05* | 0.0053 | 141 | 190 | 50 | 22 | 33 |
|  | *Syhu-DAB*05* | *Syhu-DAB*19* | 0.0126 | 141 | 190 | 50 | 22 | 31 |
|  | *Syhu-DAB*03* | *Syhu-DAB*11* | 0.0027 | 141 | 194 | 54 | 24 | 32 |
|  | *Syhu-DAB*05* | *Syhu-DAB*13* | 0.0000 | 141 | 194 | 54 | 24 | 39 |
|  | *Syhu-DAB*05* | *Syhu-DAB*11* | 0.0007 | 141 | 194 | 54 | 24 | 35 |
|  | *Syhu-DAB*07* | *Syhu-DAB*18* | 0.0004 | 143 | 180 | 38 | 18 | 44 |
|  | *Syhu-DAB*16* | *Syhu-DAB*18* | 0.0004 | 143 | 180 | 38 | 18 | 44 |
|  | *Syhu-DAB*17* | *Syhu-DAB*18* | 0.0004 | 143 | 180 | 38 | 18 | 44 |
|  | *Syhu-DAB*01* | *Syhu-DAB*18* | 0.0009 | 143 | 180 | 38 | 18 | 43 |
|  | *Syhu-DAB*14* | *Syhu-DAB*18* | 0.0009 | 143 | 180 | 38 | 18 | 43 |
|  | *Syhu-DAB*05* | *Syhu-DAB*18* | 0.0010 | 143 | 180 | 38 | 18 | 42 |
